# Effect of Nanographene Coating on the Seebeck Coefficient of Mesoporous Silicon

**DOI:** 10.3390/nano13071254

**Published:** 2023-04-01

**Authors:** Sibel Nar, Arnaud Stolz, Denis Machon, Eric Bourhis, Pascal Andreazza, Abderraouf Boucherif, Nadjib Semmar

**Affiliations:** 1 Groupe de Recherches sur l’Énergétique des Milieux Ionisés, GREMI, Université d’Orléans, CNRS, 14 Rue d’Issoudun, 45067 Orleans CEDEX 02, France; sibel.nar@usherbrooke.ca (S.N.);; 2 Laboratoire Nanotechnologies et Nanosystèmes (LN2)–CNRS IRL-3463, Université de Sherbrooke, 3000 Boulevard Université, Sherbrooke, QC J1K OA5, Canada; 3 Institut Interdisciplinaire d’Innovation Technologique (3IT), Université de Sherbrooke, 3000 Boulevard de l’Université, Sherbrooke, QC J1K OA5, Canada; 4 Université de Lyon, INSA Lyon, CNRS, École Centrale de Lyon, Université Claude Bernard Lyon 1, CPE Lyon, INL, UMR5270, 69621 Villeurbanne, France; 5 Interfaces, Confinement, Matériaux et Nanostructures, ICMN, Université d’Orléans, CNRS, 1B, Rue de la Férollerie, 45071 Orleans CEDEX 02, France

**Keywords:** mesoporous silicon, Seebeck coefficient, nanographene incorporation, electrochemical etching

## Abstract

Nanographene–mesoporous silicon (G-PSi) composites have recently emerged as a promising class of nanomaterials with tuneable physical properties. In this study, we investigated the impact of nanographene coating on the Seebeck coefficient of mesoporous silicon (PSi) obtained by varying two parameters: porosity and thickness. To achieve this, an electrochemical etching process on p + doped Si is presented for the control of the parameters (thicknesses varying from 20 to 160 µm, and a porosity close to 50%), and for nanographene incorporation through chemical vapor deposition. Raman and XPS spectroscopies confirmed the presence of nanographene on PSi. Using a homemade ZT meter, the Seebeck coefficient of the p + doped Si matrix was evaluated at close to 100 ± 15 µV/K and confirmed by UPS spectroscopy analysis. Our findings suggest that the Seebeck coefficient of the porous Si can be measured independently from that of the substrate by fitting measurements on samples with a different thickness of the porous layer. The value of the Seebeck coefficient for the porous Si is of the order of 750 ± 40 µV/K. Furthermore, the incorporation of nanographene induced a drastic decrease to approximately 120 ± 15 µV/K, a value similar to that of its silicon substrate.

## 1. Introduction

Silicon-based nanomaterials such as porous silicon have been increasingly investigation in recent years due to the properties they acquire through nanostructuring processes. These lead to significant improvements in several targeted properties, including the factor of merit (ZT) for thermoelectric concerns [1,2,3,4,5,6,7,8]. Different methods are used to nanostructure silicon substrate, from silicon nanowires [9,10] to patterned silicon thin films [11,12]. As reported in the literature, electrochemical etching is one of the easiest, fastest, and most versatile processes to achieve scalable nanostructures. These materials were first synthesized in 1956 by Uhlir et al. [13] and according to the International Union of Pure and Applied Chemistry (IUPAC), three main morphologies exist for the resulting porous layer depending on the pore diameter D_p_: microporous (D_p_ < 2 nm), mesoporous (2 nm < D_p_ < 50 nm), and macroporous (D_p_ > 50 nm). These size distributions are strongly correlated to the experimental conditions such as dopant and concentration, electrolyte composition, concentration of the hydrofluoric acid, etc. [12,13].

Microporous or macroporous morphologies are observed for low-doped p- or n-type substrates [13,14]. Highly doped silicon (with a carrier concentration from 10^19^ to 10^21^ cm^−3^) promotes a mesoporous (PSi) morphology. It demonstrates a good compromise of the three properties that are useful in thermoelectric materials, namely, the Seebeck coefficient S, which is determined by the measurement of the ratio between the induced electric potential variation (ΔU) and the temperature difference (ΔT), electrical conductivity σ (or equivalent power factor σS^2^), and thermal conductivity k [15]. In this doping range, the Seebeck coefficient values for PSi are evaluated at up to 700 µV/K versus Seebeck coefficient values of 130–170 µV/K for bulk crystalline silicon [16,17,18]. Low doping of silicon of the order of 10^18^ cm^−3^ can demonstrate a Seebeck coefficient of 660 µV/K [19] while mitigating the other parameters, leading to low ZT values.

Recent works have focused on incorporating graphene into matrices such as semiconductor ceramics [20,21]. This incorporation is interesting in combination with porous semiconductors to improve their electronic properties, especially the electrical conductivity, which is initially lowered by nanostructuring [22] without increasing the thermal conductivity. Nanographene incorporation has already been achieved due to the chemical vapor deposition (CVD) method, as recently described by Sauze et al. [23] on porous germanium and by Boucherif et al. [24] on porous silicon.

Different commercial devices called ZT meters are widely available for characterizing several kinds of materials and surfaces for S measurement. Among these devices, the two large, existing families are laser-based [25,26] and temperature gradient devices [27]. In the case of the laser devices, in view of the configuration, the measurements are made only in the plane of the surface. The use of a CO_2_ laser in the work of Melhem et al. [26] was interesting for in-plane S measurements; however, since silicon is transparent in this range, in-depth direct measurements will not be suitable. As PSi is an anisotropic layer with branched columnar pores on which surface and depth properties are different, a more specific device must be designed to determine the out-of-plane properties. Thus, a homemade setup was designed and used to obtain Seebeck measurements in both a nanographene mesoporous Si matrix (G-PSi) and PSi. In order to correlate the Seebeck behavior with the effect of the nanographene insertion, morphological, structural and chemical characterizations were systematically conducted using scanning electron microscopy (SEM) and X-ray photoelectron (XPS), ultraviolet photoelectron (UPS), and Raman spectroscopies.

## 2. Materials and Methods

### 2.1. Electochemical Etching 

**Figure 1:**
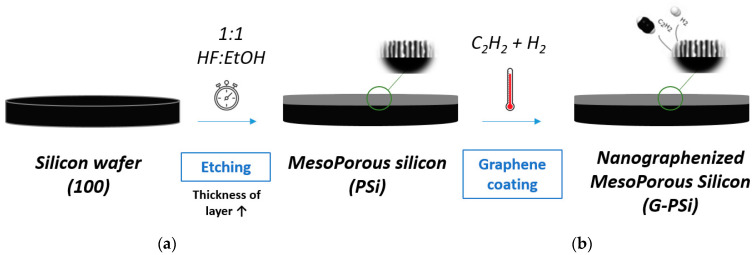
(**a**) Electrochemical etching step and (**b**) graphene coating by CVD.

Mesoporous Si samples were fabricated through the anodizing process of p-type boron-doped (100)-oriented silicon (Si) wafers (resistivity ≤ 0.005 Ω.cm) (WaferPro, Santa Clara, CA, USA). An electrochemical cell using a Si wafer as a working electrode was used to porosify the substrate. This cell was made of Teflon, with a copper electrode as the back contact of the wafer (insulated from the electrolyte) and a platinum counter electrode. The electrolyte was hydrofluoric acid (49%), it had an and ethanol in a ratio of 1:1 (*v*/*v*). The current density used was 100 mA/cm^2^. The etching was performed under a pulsed current. Between each etching sequence of 10 s, a rest time (during which no current was imposed) of 10 s was necessary to obtain thick layers. By varying the total etching duration (Figure 1a), different thicknesses of porous layers were obtained (Table 1).

### 2.2. Graphene Coating 

Using CVD, the mesoporous structure was covered by nanographene, similar to [23,24], i.e., the porous material was infiltrated by acetylene (by the addition of gas in the tubular furnace) and then annealed to 1023 K (Figure 1b). Details of the different steps are provided as supplementary information (Figure S1). This resulted in a graphene-based nanocomposite.

### 2.3. Seebeck Coeffcient Measurement using Homemade ZT Meter 

A ZT meter device waas been designed at the GREMI laboratory to determine the Seebeck coefficient of complex structures such as PSi and G-PSi. The description of our device is detailed in Section 3.4.

## 3. Results and Discussion

### 3.1. Physico-Chemical Analysis of PSi and G-PSi 

#### 3.1.1. Characterization of the Porosity in PSi

**Figure 2:**
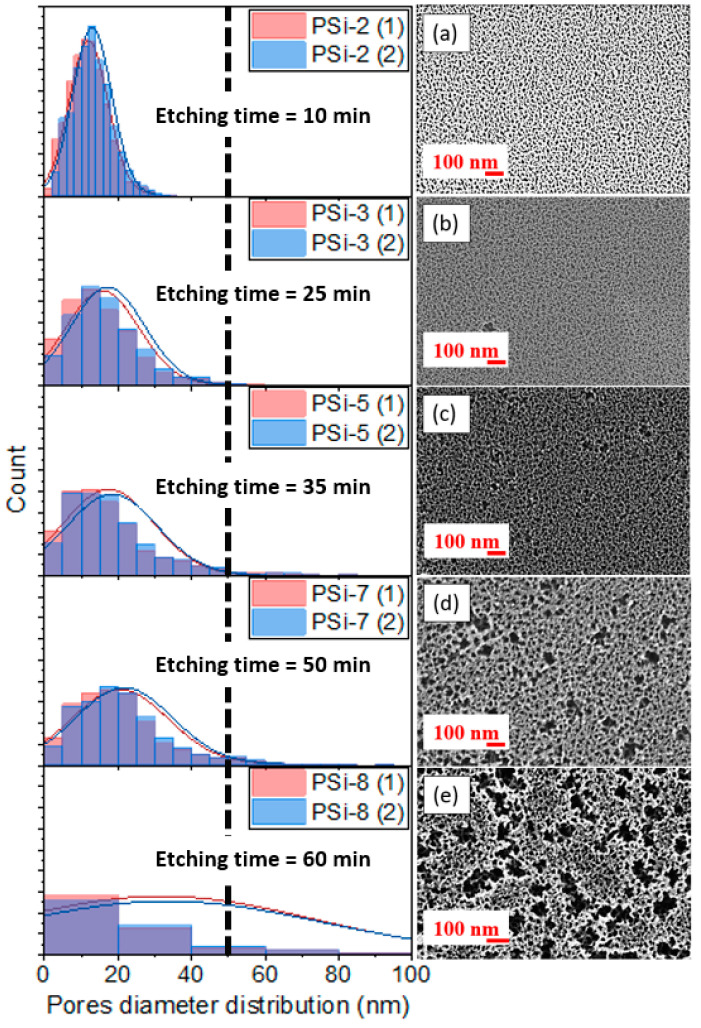
SEM images of PSi surface for different thicknesses: (**a**) PSi-2, (**b**) PSi-3, (**c**) PSi-5 (**d**) PSi-7, and (**e**) PSi-8, with a current density of 100 mA/cm^2^ and pore distribution for each sample. The limitation (dark dashes) indicates the boundary between mesopores and macropores. The Normal law representation shows the mode for each histogram. The analysis was performed twice: (1) in red and (2) in blue; both are represented for each sample.

In order to determine the porosity of the different samples, Fourier transform infrared (FTIR) spectroscopy was used according to the methodology developed by Bioud et al. [28]. The refractive index *n_p_* allows for the determination of the porosity *p* of the porous material according to:

(1) $$p=\frac{\left({n_{p}}^{2/3}-{n_{\mathrm{substrate}}}^{2/3}\right)}{\left(1-{n_{\mathrm{substrate}}}^{2/3}\right)},$$

where, in our case, *n_substrate_* = *n_silicon_* ~ 2,2 at 300 K, as cited in Ref. [29] for p-doped silicon with a dopant rate of approximately 10^20^–10^21^ cm^−3^ and at λ = 5.5 µm. The refractive index of the porous material was calculated from the FTIR spectra in the Supplementary Information (Figure S2). For instance, in the case of 30 µm, $n_{p}$ = 1.7, leading to *p* = 52%. This value of the refractive index is in perfect agreement with that provided by Canham et al. [13] and Melhem et al. [30]. This porosity calculation method was applied for all samples (PSi-1 to PSi-9), and an average porosity value of 51 ± 2% was obtained. Notice that such a porosity value is consistent for a p-type silicon under the etching conditions stated above. The second method for the determination of the porosity was to carry out ImageJ software processing. By means of image processing, we also obtained information on the distribution of pores. Figure 2 shows a typical SEM surface top view of the PSi for five porosification conditions (PSi-2, PSi-3, PSi-5, PSi-7, and PSi-8, described in Table 1).

For each PSi, size distribution analyses were carried out. Indeed, we observed that as the thickness of the porous layer increased, the number of the smallest mesopores decreased with the appearance of macropores. Specifically, from Figure 2c, the pores on surface started to widen. This widening was enhanced for a longer etching process, as reported in Figure 2d,e. Statistic results obtained for the pore distribution are summarized in Table 2.

It was therefore observed that the more the etching time increased, the more the pore density, P_d,_ decreased and the average of pore diameter, $\bar{D_{p}}$ increased. The *P_d_* was reduced by approximately three times between PSi-2 and PSi-8, and the $\bar{D_{p}}$ was multiplied by about 2.5. Melhem et al. [31] obtained a $\bar{D_{p}}$ of 10 nm and a *P_d_* of 6 × 10^3^ pores/µm^2^ for a PSi thickness of 50 µm. These values are different from those determined in our case (PSi-3). More details about error calculation are presented in the Supplementary Information (Table S1). This difference can be explained by the current density, which was 30 mA/cm^2^ in the case of Melhem et al. [31], with a 15% concentration of HF used and a substrate resistivity of 15–25 mΩ.cm. These three parameters drastically modify the characteristics of the PSi. 

Cross-sections were taken to observe the in-depth morphology. It should be noted that the corresponding morphology showed an expected columnar shape with lateral branches of the “inverted fir tree” type, which is commonly reported in the literature in the case of the electrochemical etching of p-doped silicon substrates [13].

**Figure 3:**
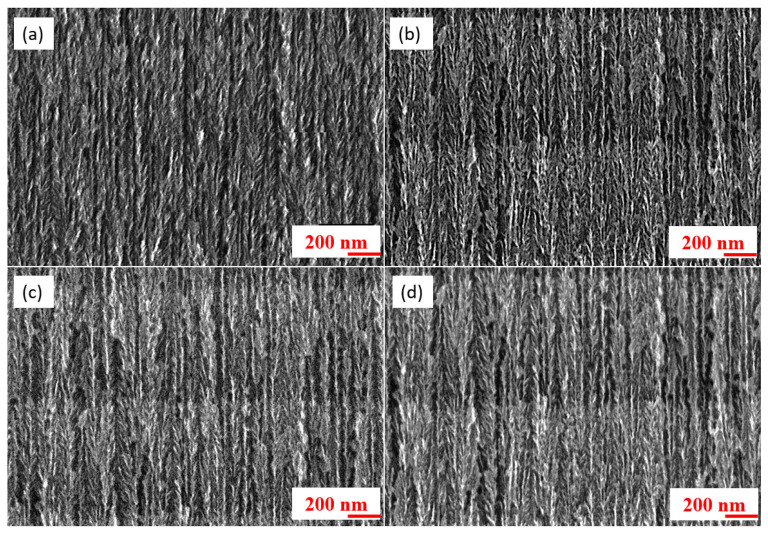
SEM images of PSi in cross-section for different thicknesses: (**a**) PSi-2, (**b**) PSi-3, (**c**) PSi-5, and (**d**) PSi-8 with a current density of 100 mA/cm for 10 s etching pulse duration.

**Figure 4:**
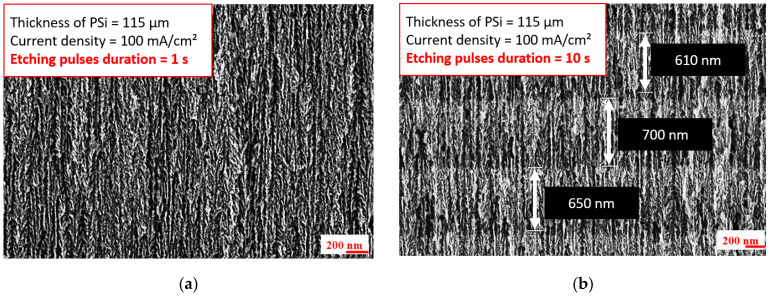
Focus on modulation visible in-depth for samples with a thickness of 115 µm under different pulse duration conditions with 30,000× magnification and 15 kV: (**a**) 1 s and (**b**) 10 s.

Figure 3 shows four cross-sectional images of PSi. Unlike the top view SEM images, the cross-sectional structures do not show a significant difference in morphology or pore size. However, several porosity modulations are visible and they are especially more apparent in sections with high porous thicknesses (Figure 3c,d). Electrochemical etching is performed in two steps: an etching pulse and a passivation pulse. The imposed current pulses are identical, and both last equal 10 s. During the passivation pulses, no current is injected. The duration of these pulses therefore affects the diameter of the pores. The origin of the modulations was investigated by varying the pulse time. The SEM images in cross-section were obtained in 1 s (Figure 4a) and 10 s pulses (Figure 4b).

When using a pulse time of 10 s, the modulations were very regular and were approximately between 600 and 700 nm in size (Figure 4b). In the case of the 1 s etching pulses (Figure 4a), for the same thickness obtained, there was no apparent modulation contrary to the 10 s pulses. Therefore, the appearance of these modulations can be explained by the etching cycle. Kuntyi et al. [32] also underlined that the pore geometry and the architecture of the PSi vary according to several parameters, including the current regime and the total anodization time. This indicates that for a pulsed current etching regime, the duration of the imposed pulses will impact the formed porous structure. A high current flow can cause tetravalent dissolution, as indicated in Ref. [13], and these conditions correspond to the electropolishing of silicon, involving the formation of an oxide. Low current densities and etching times are necessary to remain in the porous formation zone. By imposing 10 s pulses with long etching times, as in Figure 2d,e, we find ourselves in a transition regime between the formation of pores and electropolishing, when there is no longer any nanostructuring. These periodic modulations can affect in-depth porosity of the PSi. Lascaud et al. [33] showed that it is necessary to adjust the current density to control the in-depth porosity in the case of high, thick layers of PSi obtained from a p+ type substrate.

#### 3.1.2. Porosity in G-PSi

As the CVD is carried out at a high temperature (750 °C or 1023 K), it is also necessary to characterise the G-PSi in a cross-section to verify if there is any redensification of the PSi caused by the graphene insertion. 

**Figure 5:**
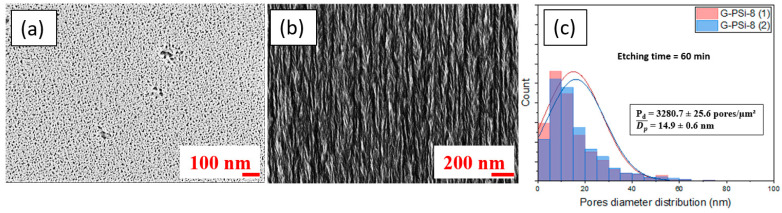
Characterization of G-PSi: (**a**) SEM top surface and (**b**) cross-section with (**c**) pore diameter distribution for G-PSi-8.

Figure 5 shows SEM images for the G-PSi-8, obtained under the same electrochemical etching conditions as the PSi-8 (condition Figure 2e). In comparing the PSi-8 and G-PSi-8, we note that the pore density was greater after the CVD, increasing from 1547.3 to 3280.7 pores/µm^2^. Additionally, the average pore diameter was divided by two, and was 14.9 nm for the G-PSi-8. Thus, it can be seen in Figure 5b that the mesoporous structure of G-PSi was retained. The CVD was then realized without significant modification of the tree-like structure. The contrast of the two images (Figure 3d and Figure 5b) is probably linked to the presence of carbon in the case of G-PSi due to its higher electronic conduction than PSi.

After the microscopic analyses of the G-PSi surface and cross-section, the nature and quality of the carbon infiltrated in the mesoporous structure was investigated using Raman and XPS spectroscopic analyses.

### 3.2. Raman and XPS Analyses

**Figure 6:**
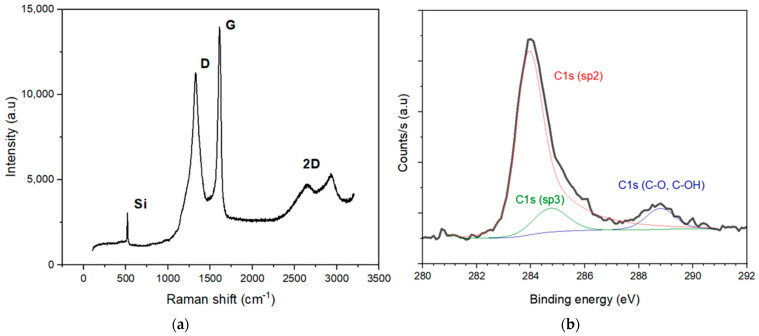
Spectroscopy analyses: (**a**) Raman spectrum of G-PSi-8 and (**b**) XPS analysis of G-PSi-8.

The carbon coating was systematically characterized by Raman spectroscopy. A StellarNet Raman spectroscope was used, consisting of a visible laser with a wavelength of λ_L_ = 532 nm and a power of 100 mW. The integration time was 10 s. Figure 6a shows the Raman spectrum. The Raman spectrometric analysis was performed on all G-PSi samples. Firstly, The D and G peaks located at 1327 cm^−1^ and 1603 cm^−1^, respectively, can be considered a signature of carbon [34]. The Raman spectrum of graphite contains two peaks, therefore the G is found at around 1580 and 1600 and a D peak is found around 1350 cm^−1^ [35]. The presence of defects within the disordered material is responsible for the D peak. In addition, the 2D peak has a very low intensity with a larger width compared to the two peaks. It has been shown that at higher synthesis temperatures, this disorder can decrease [36]. By determining the ratio I(D)/I(G), it is possible to extract information such as the size of the domains in the coating layer. According to the work of Ferrari and Robertson [35] and Sauze et al. [23], the carbon structure incorporated into the porous material is called a “nanographene”. [23,35,37]. Tuinstra and Koenig’s relationship explains that the ratio of the intensities of the D and G peaks is inversely proportional to the size of the domain [34,35,38,39], as:

(2) $$\frac{I(D)}{I(G)}=\frac{C(\lambda )}{La},$$

where C (λ = 532 nm) ~ 49.6 Å [34]. A measured I(D)/I(G) ~ 0.8–0.9 leads to a domain size of graphene La of 5.5–6.2 nm for every G-PSi sample. Pure graphene has excellent electronic properties and thermal properties as well, a property unsuitable for thermoelectricity. The quality of our coating is sufficient to evaluate the impact of G-PSi in the Seebeck coefficient of the final nanomaterial. It is interesting to note that the representative peak of Si-C at 970 cm^−1^ was absent in the Raman spectrum, indicating the absence of SiC formation.

XPS analyses were carried out to provide information on the presence of an oxide on the surface after graphene deposition. The XPS analysis (monochromatic source, KαAl (1486.6 eV), with a detection energy resolution of less than 0.1 eV) was performed on five different points. A representative spectrum is presented in Figure 6b. All the spectra show the same trend. First, it shows that the carbon coating is homogeneous. 

Secondly, it is possible to affirm that we observed a nanographene with the presence of C-sp3 and C-sp2 bonds. This observation was also highlighted by Sauze et al. in the case of nanographenized porous germanium, for which the insertion of nanographene was also carried out by CVD at different temperatures [23]. The analysis also shows that the nanographene surface was oxidized with the presence of a peak that was related to C-O and C-OH.

### 3.3. UPS Analysis for Seebeck Determination

**Figure 7:**
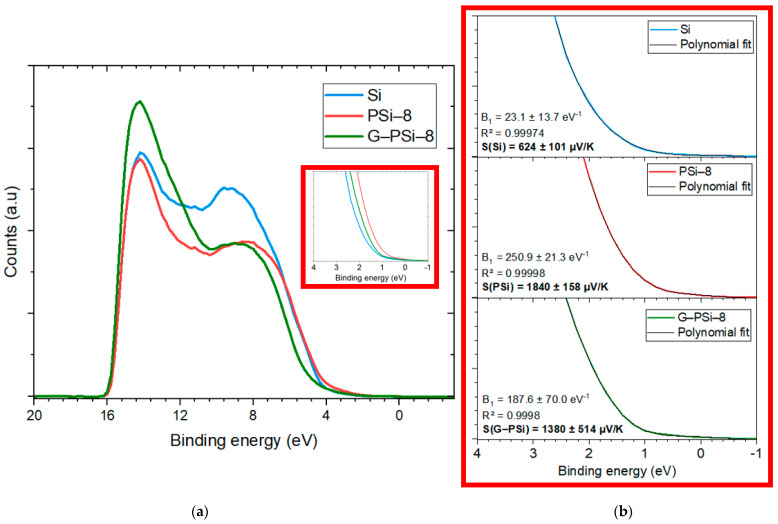
(**a**) UPS spectra of Si, PSi-8, and G-PSi-8; (**b**) zoom on lower binding energy (E_B_): E_B_ equal to 0 represents the Fermi level.

Ultraviolet photoelectron spectroscopy (UPS) is a technique for probing the valence electronic states in semiconductor materials. Therefore, the Seebeck coefficient of a material can be determine using this technique [40]. Several works have presented UPS as a relevant characterization technique, especially for graphene [41,42,43]. Then, according to the complexity of the PSi material, with marked anisotropy and branching columnar pores formed deeply in the layer, we used this spectroscopic method for S determination without any contact. It is a surface analysis technique (below 10 nm of thickness) for the determination of the electrical properties of a semiconductor; for example, the Fermi level and work function, which depends on doping. The Seebeck coefficient S is related to the entropy per charge carrier [44]. Mott’s formula describes that S is linked to the density of state (DoS) and can be expressed as:

(3) $$S\left(E,T\right)=\frac{\pi^{2}}{3}\cdot \frac{{k_{B}}^{2}T}{q}\cdot {\left(\frac{\partial ln(\sigma \left(E\right))}{\partial E}\right)}_{E=E_{F}},$$

where S is the Seebeck coefficient, k_B_ is the Boltzmann constant, q is the elementary charge, T is the temperature, E is the binding energy, E_F_ is the energy of Fermi level, and σ is the electrical conductivity. The Seebeck coefficient is directly proportional to the derivative of DoS, which depends on the energy [45]. The DoS is the number of electronic states that are available in a system per unit volume and energy intervals. The term ∂ln(σ(E))/∂E is linked to the shape of the DoS at the Fermi level and describes its tangent at this level because σ(E) depends on N (charge carrier density) which is linked to D(E), the number of available states that an electron can occupy. The UPS measurements were performed with a ThermoScientific Escalab Xi+ as XPS analyses. This photoelectron spectrometer is equipped with a UV source of He I with an energy of 21.2 eV. The size of the analysis area is 650 µm. UPS spectra for Si, P-Si-8, and G-PSi-8 are provided in Figure 7.

Based on Mott’s formula, it is possible to compare the S of different materials. In the works of Perrot et al. [46], the comparison of the S of two semi-metallic polymer materials was qualitative. In our work, to quantify S, the spectra were fitted by a polynomial function of the order 5, type y= A+ B_1×_+B_2×_^2^+B_3×_^3^+B_4×_^4^+B_5×_^5^, and selected with quality criteria R^2^ > 0.999 and a B_1_ value. More details concerning the fit are presented in the Supplementary Information (Figure S3 and Table S2). This function was derived to obtained ∂ln(σ(E))/∂E at x = 0 (B_1_ on Figure 7). The different values obtained are presented in insert of Figure 7b. S is determined by Equation (3).

Through UPS, the calculation of S (Si) provided a value S(Si) = 170 ± 101 μV/K. Therefore, the in-plane S(Si) takes values in the range of 69–271 µV/K, values which are in good agreement with Refs. [16,17,18], which reported S(Si) values between 130–170 µV/K for a dopant rate of around 10^20^ cm^−3^ from a determination was made using a conventional method of measuring in-plane S. On the other hand, the S of the PSi is clearly higher than indicated in the literature, which provides values of S(PSi) equal to 1014 µV/K for a maximum value [27]. UPS is a surface analysis, and the surface of PSi oxidizes easily after exposure to ambient air. It is also reported in the literature that oxide surfaces have a high Seebeck coefficient of about 1000 µV/K at 340 K [47]. The in-plane S determination with the oxide overestimated the value of the S(PSi). However, we can affirm that the S of PSi is greater than S(Si) and S(G-PSi). For G-PSi, the literature reports no result on S measurements to confirm this value. Given the complexity of the material, we can say that the value is probably also overestimated, similar to PSi. Equation (4) depends on two components, mobility and the shape of the DoS [44,45], and we can write:

(4) $$S\left(E,T\right)=\frac{\pi^{2}}{3}\cdot \frac{{k_{B}}^{2}T}{q}\cdot {\left(\frac{1}{n}\frac{dn(E)}{dE}+\frac{1}{µ}\frac{dµ(E)}{dE}\right)}_{E=E_{F}},$$

where the terms n(E) and µ(E) are the energy-dependent carrier density (affect DoS) and mobility (affect electronic properties), respectively. Then, we can assume that similar to Si, G-PSi is more conductive than Psi, so we increase mobility-dependent part. For PSi, the DoS dependent component is certainly higher, contrary to the mobility-dependent part, which demonstrates a high S. In contrast, G-PSi is normally more conductive than PSi [48] because there is nanographene in its pores; this affects the DoS-related component differently than Psi, which has empty pores. In this case, both components (DoS and mobility) affect the S. Indeed, the error calculated by UPS on S is smaller on Si than on PSi and G-PSi. Again, this is likely related to the porous, complex structure of PSi and G-PSi.

Given the high values of the S of PSi and probably for G-PSi by UPS, macroscopic S measurement are needed in order to verify the S of our materials in depth. A device adapted to the measurements of S of the PSi and G-PSi was developed for the in-depth S determination of this complex structure.

### 3.4. ZT Meter Design for Seebeck Measurement

**Figure 8:**
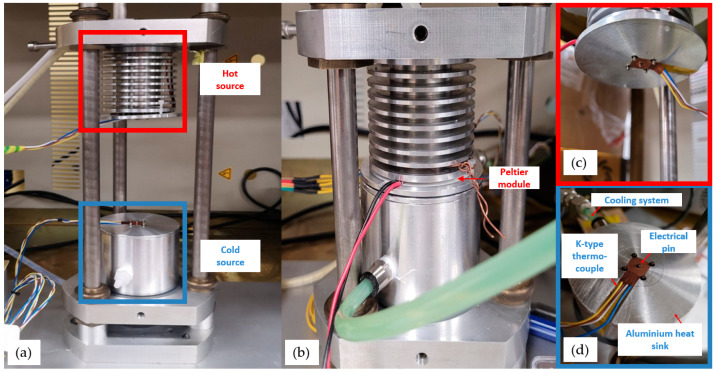
(**a**) Homemade ZT meter device for in-depth S measurement: view before measurement; (**b**) closed system with a mobile upper part: view during measurement; (**c**) zoom on hot block; (**d**) zoom on cold block with integrated sensors description.

The homemade device was based on the application of a temperature gradient and ia composed of two aluminium heat sinks (a hot source at the top and a cold source at the bottom) arranged on a vertical system (Figure 8a,b). It allowed the thermal conductivity and the Seebeck coefficient to be measured simultaneously. As shown in Figure 8c, the hot source was equipped on its surface with a Peltier module to create the temperature rise and contained a suitable thermal sensor (heat flux and temperature), which also included electrical pins (spring pins facilitating surface contact). The cold source was also equipped with a thermal sensor and was then connected to a cryostat, which ensured the cooling of the entire cold block (Figure 8d). The sample was trapped between the hot and cold sources and was in direct contact with the temperature sensors on both sides, as illustrated on Figure 8. The electrical and thermal sensors were connected to a Keithley DAQ6510 instrument, which provided data acquisition of the electrical potential and temperature difference values through KickStart^®^ software (Beaverton, OR, USA).

**Figure 9:**
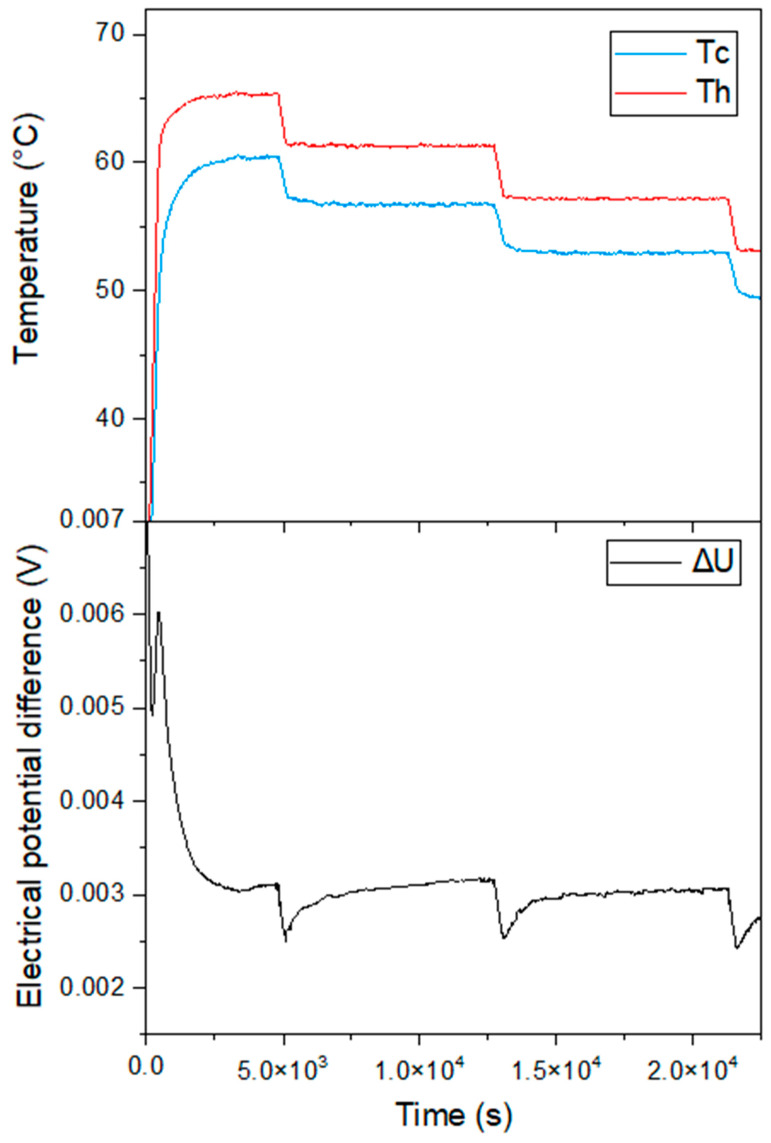
Example of cooling sequence for establishing ΔT in a temperature range from 70 to 50 °C for PSi-7 and measurement of ΔU.

By varying the temperature of the cryostat, different temperatures from the hot source T_h_ and the cold source T_c_ were therefore obtained. Data acquisition was carried out and monitored in real time. Several parameters were recorded including the different acquisition times, and the potential difference (ΔU) and different temperatures measured. The total recording time was the same for each sample. To evaluate S, a heating sequence was performed over a temperature range from 30 to 70 °C. Once T_c_ and T_h_ were stabilized, the value of ΔT (T_h_ − T_c_) was determined and then ΔU was determined, as shown in Figure 9.

This method was first carried out for bulk materials, bismuth tellurium (Bi_2_Te_3_), copper (Cu), and Si. Their characteristics and S values are presented in Table 3 and in the Supplementary Information (Figure S4). For Si, as a further precaution, a thin film of platinum (~5 nm) was deposited by evaporation on bulk silicon in order to confirm that the Seebeck value was not impacted by metal–semiconductor contacts. After the calibration, the measurements are carried out on PSi and G-PSi. In order to properly measure the S of our materials, the same deoxidation method was performed on all samples before each measurement.

### 3.5. Seebeck Coeffcient Values of PSi and G-PSi

**Figure 10:**
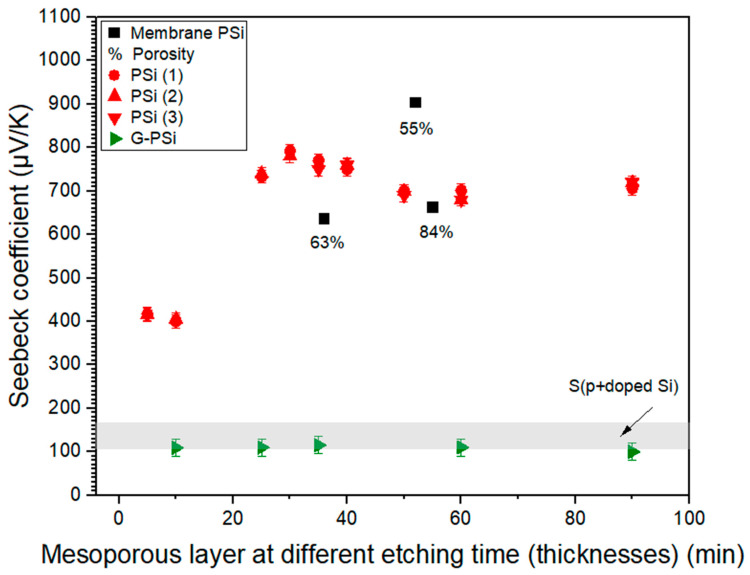
Seebeck coefficient of p + doped Si (our work and [16,17,18]), Psi, and G-PSi at T = 343 K. By increasing the electrochemical etching time, the thickness of the mesoporous layer was increased with a fixed current density of 100 mA/cm^2^, which has a weak impact on the porosity. On the other hand, the values of the membrane PSi and porosity [27] were obtained at different etching times and current density, which explains the large variation in porosity.

Figure 10 presents the S values of PSi/G-Psi, measured for different porous layer thicknesses. The S value of Si [16,17,18] are provided as a reference. Table 3 also summarizes the characteristics of the materials, including the doping type and the S.

First, as detailed in Figure 10 from (1) to (3), the measurements were repeated three times for each PSi sample. According to our results, we can affirm that we obtained an in-depth S(PSi) = 750 ± 40 µV/K, for which S was independent of the electrochemical etching process, from an etching time of 25 min (55 µm). The first two values measured, respectively, S = 417 μV/K and S = 400 μV/K, were approximately two times lower than the optimal S(PSi) measured. Indeed, S is an intrinsic property of the material, independent of the thickness. The variations in the thicknesses of the PSi layers are of particular interest in this study to overcome the contribution of the Si substrate. Indeed, due to its higher electrical conduction, Si, is conversely proportional to S and can cause a decrease in the S measured. In order to verify this hypothesis, a simulation was carried out by COMSOL Multiphysics^®^ (Grenoble, France). The results are presented in the Supplementary Materials (Figure S5 with Refs. [54,55]). It was therefore observed that the thermal gradient takes place essentially in the mesoporous layer, and that the contribution of Si substrate in the measurement of S(PSi) is made even lower by increasing the thickness of the mesoporous layer.

In Figure 2 and Figure 4, we observed different pore size distributions and a variation in pore density that correlated with the pulse/etching process duration. The enlargement of the surface pores and the variation in porosity linked to the etching pulses and the modulations can also affect the measurement of S. Valalaki et al. [27] showed variations of S related to different porosities, as shown in Figure 10. For a porosity value of 63% and a thickness of 36 µm, the S was approximately 600 µV/K. However, for a porosity of 55% and a PSi thickness of 52 µm, S = 900 µV/K. Therefore, an 8% variation in surface porosity implies an increase of S of about 300 µV/K. The surface porosity greatly affected the value of the in-plane S measured. The works of Melhem et al. [31] showed that for p-doped silicon with a crystal orientation of (100), although the current density was fixed at 30 mA/cm^2^, changes in terms of porosity were also noted for same thickness range. According to the surface of our mesoporous layers and process parameters, we can assume variations in surface and in-depth porosity. This was shown by Melhem et al. [31] in reflectivity analyses. Indeed, the depth porosity also varies according to the evolution of the dielectric functions reducing the average density of the Si-Si bonds.

In another work, Melhem et al. [30] observed through transmission electron microscopy that the crystallinity of silicon is preserved with an oxidized amorphous layer along the pores. As discussed in Section 3.3, the presence of these crystalline and amorphous phases affects the Seebeck coefficient of semiconductor materials and semi-metals. Also, their work on optical characterization showed an evolution of the optical response of the PSi that originated from the structural and chemical evolution of the porous layers, with porosities ranging from 28 to 41% and thicknesses ranging from 0.2 to 50 µm, approximately. We can therefore consider that the chemical structure of the PSi evolves with further etching and the presence of Si-H and Si-O links [30,31,56,57], which affects the transport properties. These authors showed that the period and the amplitude of the interferential oscillations in the layers reveal a dependence on the porosity and the thickness of the PSi. Indeed, S depends on carrier diffusion (S_d_) and phonon drag (S_ph_). In nanostructured semiconductors, both effects have an impact on the variation of the total S. This low dimensionality causes quantum confinement, which impacts the transport of charge carriers. In the work of Singh et al. [58], we observed that the S of silicon nanocrystals (Si NCs) was lower for a size of 2.4 nm, unlike the S obtained for larger sizes of NC of 5.6 and 8.3 nm. Analyses of the coupling constants showed that the coupling of acoustic phonons is approximately three times higher in the 2.4 nm NC, unlike the 5.6 and 8.3 nm NC. These deviations therefore affect the dynamics and the diffusion of the carriers as well as the thermoelectric properties of the nanostructured silicon. Due to strong phonon scattering in small NCs and the likely reduction of phonon lifetime (τ_ph_) with NC size (S_ph_ ~ τ_ph_/μT, with μ is the electron mobility) [57,58,59], the strong coupling of electrons with phonons leads to much shorter carrier diffusion and phonon drag, and a strong reduction in S observed in small Si NCs.

In the case of G-PSi, unlike PSi, the S = 120 ± 15 µV/K measured for each sample is very close to S(Si). Nanographene is as good an electrical conductor as Si. This feature assigns other properties, including S, which is affected by electron mobility. We can assume that the presence of carbon makes it possible to stabilize the mesoporous structure and drastically reduce the S, which is very close to that of the Si substrate. Contrary to the values obtained by UPS, the in-depth S (PSi) and (G-PSi) are lower. Comparing these results, the S(Si) values are closely similar with the S(Si)= 170 ± 101 µV/K and 100 ± 15 µV/K, obtained by UPS and ZT meter, respectively. Keeping in mind that the UPS method concerns the in-plane S values, a significant difference is noted for S(PSi) and S(G-PSi) values. Additional investigations on thermal and electrical conductivities are in progress to evaluate the thermoelectric efficiency of nanocomposites.

## 4. Conclusions

In this work, we investigated the relation between a nanographene-coated and an uncoated porous structure and the Seebeck coefficient. First, we successfully measured the S(PSi) = 750 ± 40 µV/K, which is uncorrelated to the mesoporous thickness and to the Si sublayer and is more conductive than nanostructured Si. Moreover, Raman, XPS, and UPS spectroscopy analyses confirmed the presence of nanographene but also of some amorphous phases and oxidized surfaces that explain the higher S values compared to the p+ doped Si in the case of PSi and G-PSi. In conclusion, the unexpected effect is induced by the nanographene, which contributes to a higher electronic transport but a lower Seebeck value, close to S(Si) values, contrary to the trends investigated by UPS.

## Figures and Tables

**Table 1:** Electrochemical etching process characteristics.

Etching Duration (min)	Mesoporous Layer Thicknesses (µm)	Porosity by FTIR (%)	Porosity by SEM (%)	Sample
0	0	0	0	Si
5	20	51 ± 1	51 ± 1	PSi-1
10	30	52 ± 2	49 ± 1	PSi-2
25	55	53 ± 3	50 ± 1	PSi-3
30	60	53 ± 3	52 ± 2	PSi-4
35	70	50 ± 1	50 ± 1	PSi-5
40	90	52 ± 2	51 ± 1	PSi-6
50	115	50 ± 1	49 ± 1	PSi-7
60	135	49 ± 1	55 ± 5	PSi-8
90	160	49 ± 1	54 ± 4	PSi-9
10	30	-	47 ± 3	G-PSi-2
25	55	-	47 ± 3	G-PSi-3
35	70	-	48 ± 2	G-PSi-5
60	135	-	49 ± 1	G-PSi-8
90	160	-	51 ± 1	G-PSi-9

**Table 2:** Pore distribution analysis in PSi.

Sample	Pores Density (pores/µm^2^) $\left(P_{d}\right)$	Average of Pores Diameter (nm) $\left(\overset{-}{D_{p}}\right)$
PSi-2	4376.7 ± 21.4	12.0 ± 0.5
PSi-3	3284.7 ± 18.2	15.7 ± 0.6
PSi-5	2873.6 ± 17.5	17.4 ± 0.6
PSi-7	2439.0 ± 13.3	20.5 ± 1.5
PSi-8	1547.3 ± 10.8	25.5 ± 1.9

**Table 3:** Summary of materials characteristics.

Material	Dopant	Thickness (µm)	Dopant Rate (cm^−3^)	S (µV/K)
Bi_2_Te_3_	N	3000	10^18^	−175 ± 10 (T = 350 K)
Bi_2_Te_3_ [49]	N	2000	not indicated	−140 (T = 350 K)
Bi_2_Te_3_ [50]	N	not indicated	not indicated	−160 (T = 325 K)
Bi_2_Te_3_ [51]	N	not indicated	3–4 × 10^19^	−175 (T = 350 K)
Cu	undoped	450	undoped	1.5 ± 0.5
Cu [52]	undoped	not indicated	undoped	2–3
Cu [53]	undoped	not indicated	undoped	2 ± 1
Si	P/p+	500	10^20^—10^21^	100 ± 15
Si [18]	P/p+	not indicated	1.2 × 10^20^	170
Si [17]	P/p+	not indicated	10^20^–10^21^	150
Si [16]	P/p+	not indicated	10^20^–10^21^	130
PSi-1–PSi-9	P	20–160	-	400 ± 15 – 792 ± 15
PSi [27]	P	120	-	917
PSi [27]	P	100	-	903
PSi [27]	P	100	-	636
G-PSi-2–G-PSi-9	P	30–160	-	110 ± 15 –125 ± 15

## Data Availability

The data are included in the main text and in the Supplementary Information.

## Supplementary Materials

The following supporting information can be downloaded at: https://www.mdpi.com/article/10.3390/nano13071254/s1. Figure S1: Steps of CVD for graphene coating of PSi; Figure S2: Refractive index determination of PSi by FTIR (red line for PSi-2 and blue line for PSi-3); Table S1: Calculation of deviation and errors; Figure S3: Methodology for fit and S value determination of Si by UPS; Table S2: Summary of polynomial fit characteristics; Figure S4: S values for Si, Cu, and Bi_2_Te_3_; Figure S5: (a) System studied under COMSOL Multiphysics ^®^; (b) Establishment of the thermal gradient in the PSi and Si. The insert PSi thicknesses were selected as an example for the simulation study.

## References

[1] Halimaoui A. (1994). Determination of the specific surface area of porous silicon from its etch rate in HF solutions. Surf. Sci..

[2] Amin-Chalhoub E., Semmar N., Coudron L., Gautier G., Boulmer-Leborgne C., Petit A., Gaillard M., Mathias J., Millon E. (2011). Thermal conductivity measurement of porous silicon by the pulsed-photothermal method. J. Phys. D Appl. Phys..

[3] Acquaroli L.N., Brondino A., Schmidt J.A., Arce R.D., Koropecki R.R. (2009). Infrared study of the oxidation of porous silicon: Evidence of surface modes. Phys. Status Solidi C.

[4] Dhanekar S., Jain S. (2013). Porous silicon biosensor: Current status. Biosens. Bioelectron..

[5] Rauscher M., Spohn H. (2001). Porous silicon formation and electropolishing. Phys. Rev. E.

[6] Minnich A.J., Dresselhaus M.S., Ren Z.F., Chen G. (2009). Bulk nanostructured thermoelectric materials: Current research and future prospects. Energy Environ. Sci..

[7] Dresselhaus M.S., Chen G., Tang M., Yang R., Lee H., Wang D., Ren Z., Fleurial J., Gogna P. (2006). New Directions for Nanoscale Thermoelectric Materials Research. MRS Online Proc. Libr..

[8] Dresselhaus M.S., Chen G., Tang M., Yang R., Lee H., Wang D., Ren Z., Fleurial J., Gogna P. (2007). New Directions for Low-Dimensional Thermoelectric Materials. Adv. Mater..

[9] Hippalgaonkar K., Huang B., Chen R., Sawyer K., Ercius P., Majumdar A. (2010). Fabrication of microdevices with integrated nanowires for investigating low-dimensional phonon transport. Nano Lett..

[10] De Boor J., Geyer N., Wittemann J.V., Gösele U., Schmidt V. (2010). Sub-100 nm silicon nanowires by laser interference lithography and metal-assisted etching. Nanotechnolog.

[11] Tang J., Wang H.T., Lee D.H., Fardy M., Huo Z., Russell T.P., Yang P. (2010). Holey Silicon as an Efficient Thermoelectric Material. Nano Lett..

[12] Yu J.K., Mitrovic S., Tham D., Varghese J., Heath J.R. (2010). Reduction of thermal conductivity in phononic nanomesh structures. Nat. Nanotechnol..

[13] Canham L. (2014). Handbook of Porous Silicon.

[14] Gautier G., Kouassi S. (2015). Integration of porous silicon in microfuel cells: A review. Int. J. Energy Res..

[15] Godart C. (2009). Aspects Théoriques en Thermoélectricité. Techniques de l’Ingénieur.

[16] Nadtochiy A., Kuryliuk V., Strelchuk V., Korotchenkov O., Li P.W., Lee S.W. (2019). Enhancing the Seebeck effect in Ge/Si through the combination of interfacial design features. Sci. Rep..

[17] Ikeda H., Salleh F. (2010). Influence of heavy doping on Seebeck coefficient in silicon-on-insulator. Appl. Phys. Lett..

[18] Geballe T.H., Hull G.W. (1955). Seebeck Effect in Silicon. Phys. Rev..

[19] Ohishi Y., Xie J., Miyazaki Y., Aikebaier Y., Muta H., Kurosaki K., Yamanaka S., Uchida N., Tada T. (2015). Thermoelectric properties of heavily boron- and phosphorus-doped silicon. Jpn. J. Appl. Phys..

[20] Srivastava D., Norman C., Azough F., Ekren D., Chen K., Reece M.J., Kinloch I.A., Freer R. (2019). Anisotropy and enhancement of thermoelectric performance of Sr _0.8_ La _0.067_ Ti _0.8_ Nb _0.2_ O _3−δ_ ceramics by graphene additions. J. Mater. Chem. A.

[21] Ekren D., Cao J., Azough F., Kepaptsoglou D., Ramasse Q., Kinloch I.A., Freer R. (2022). Controlling the Thermoelectric Behavior of La-Doped SrTiO_3_ through Processing and Addition of Graphene Oxide. ACS Appl. Mater. Interfaces.

[22] Khardani M., Bouaïcha M., Dimassi W., Zribi M., Aouida S., Bessaïs B. (2006). Electrical conductivity of free-standing mesoporous silicon thin films. Thin Solid Films.

[23] Sauze S., Aziziyan M., Brault P., Kolhatkar G., Ruediger A., Korinek A., Machon D., Arès R., Boucherif A. (2020). Integration of 3D nanographene into mesoporous germanium. Nanoscale.

[24] Boucherif A.R., Boucherif A., Kolhatkar G., Ruediger A., Arès R. (2017). Graphene-Mesoporous Si Nanocomposite as a Compliant Substrate for Heteroepitaxy. Small.

[25] Linseis V., Völklein F., Reith H., Nielsch K., Woias P. (2018). Advanced platform for the in-plane *ZT* measurement of thin films. Rev. Sci. Instrum..

[26] Melhem A., Rogé V., Dai Huynh T.T., Stolz A., Talbi A., Tchiffo-Tameko C., Lecas T., Boulmer-Leborgne C., Millon E., Semmar N. (2018). Laser-based setup for simultaneous measurement of the Seebeck coefficient and electrical conductivity for bulk and thin film thermoelectrics. Rev. Sci. Instrum..

[27] Valalaki K., Benech P., Nassiopoulou A.G. (2016). High Seebeck Coefficient of Porous Silicon: Study of the Porosity Dependence. Nanoscale Res. Lett..

[28] Bioud Y.A., Boucherif A., Belarouci A., Paradis E., Fafard S., Aimez V., Drouin D., Arès R. (2017). Fast growth synthesis of mesoporous germanium films by high frequency bipolar electrochemical etching. Electrochim. Acta.

[29] Basu S., Lee B.J., Zhang Z.M. (2010). Infrared Radiative Properties of Heavily Doped Silicon at Room Temperature. J. Heat Transf..

[30] Melhem A., Defforge T., Meneses D.S., Andreazza-Vignolle C., Gautier G., Semmar N. (2015). Structural, Optical, and Thermal Analysis of n-Type Mesoporous Silicon Prepared by Electrochemical Etching. J. Phys. Chem. C.

[31] Melhem A., Meneses D.S., Andreazza-Vignolle C., Defforge T., Gautier G., Sauldubois A., Semmmar N. (2017). Structural, Optical, and Thermophysical Properties of Mesoporous Silicon Layers: Influence of Substrate Characteristics. J. Phys. Chem. C.

[32] Kuntyi O., Zozulya G., Shepida M. (2022). Porous Silicon Formation by Electrochemical Etching. Adv. Mater. Sci. Eng..

[33] Lascaud J., Defforge T., Certon D., Valente D., Gautier G. (2017). In-depth porosity control of mesoporous silicon layers by an anodization current adjustment. J. Appl. Phys..

[34] Ferrari A.C., Meyer J.C., Sscardaci C., Casiraghi C., Lazzeri M., Mauri M., Piscanec S., Jiang D., Novoselov K.S., Roth S. (2006). Raman spectrum of graphene and graphene layers. Phys. Rev. Lett..

[35] Ferrari A.C., Robertson J. (2000). Interpretation of Raman spectra of disordered and amorphous carbon. Phys. Rev. B.

[36] Merlen A., Buijnsters J.G., Pardanaud C. (2017). A guide to and review of the use of Multiwavelength Raman Spectroscopy for characterizing defective aromatic carbon solids: From graphene to amorphous carbons. Coatings.

[37] Novoselov K.S., Geim A.K., Morozov S.V., Jiang D., Zhang Y., Dubonos S.V., Grigorieva I.V., Firsov A.A. (2004). Electric field effect in atomically thin carbon films. Science.

[38] Tuinstra F., Koenig J.L. (1970). Raman Spectrum of Graphite. J. Chem. Phys..

[39] Matthews M.J., Pimenta M.A., Dresselhaus G., Dresselhaus M.S., Endo M. (1999). Origin of dispersive effects of the Raman D band in carbon materials. Phys. Rev. B.

[40] Petsagkourakis I., Pavlopoulou E., Cloutet E., Chen Y.F., Liu X., Fahlman M., Berggren M., Crispin X., Dilhaire S., Fleury G. (2018). Correlating the Seebeck coefficient of thermoelectric polymer thin films to their charge transport mechanism. Org. Electron..

[41] Chien C.S.C., Chang H.M., Lee W.T., Tang M.R., Wu C.H., Lee S.C. (2017). High performance MoS_2_ TFT using graphene contact first process. AIP Adv..

[42] Zhang F., Klein C., Longhi E., Barlow S., Marder S.R., Sarusi G., Kahn A. (2019). Molecular-Reductant-Induced Control of a Graphene–Organic Interface for Electron Injection. Chem. Mater..

[43] Al-Gaashani R., Najjar A., Zakaria Y., Mansour S., Atieh M.A. (2019). XPS and structural studies of high quality graphene oxide and reduced graphene oxide prepared by different chemical oxidation methods. Ceram. Int..

[44] Wang Y., Hu Y.J., Bocklund B., Shang S.L., Zhou B.C., Liu Z.K., Chen L.Q. (2018). First-principles thermodynamic theory of Seebeck coefficients. Phys. Rev. B.

[45] Jonson M., Mahan G.D. (1980). Mott’s formula for the thermopower and the Wiedemann-Franz law. Phys. Rev. B.

[46] Perrot S. (2021). Semi-Metallic Polymers for Thermoelectric Applications. Ph.D. Thesis.

[47] Mulenko S.A., Stefan N., Len E.G., Skoryk M.A., Popov V.M., Gudymenko O.Y. (2021). Laser synthesis of copper oxides 2D structures with high Seebeck coefficient and high thermoelectric figure of merit. J. Mater Sci. Mater. Electron..

[48] Sauze S. (2021). Synthèse et Caractérisation de Nanocomposites à Base de Silicium ou de Germanium Mésoporeux Carbonisés pour des Applications Thermoélectriques. Ph.D. Thesis.

[49] Wu F., Song H., Jia J., Hu X. (2013). Effects of Ce, Y, and Sm doping on the thermoelectric properties of Bi2Te3 alloy. Prog. Nat. Sci. Mater. Int..

[50] Sun M., Zhang P., Li Q., Tang G., Zhang T., Chen D., Qian Q. (2022). Enhanced N-Type Bismuth-Telluride-Based Thermoelectric Fibers via Thermal Drawing and Bridgman Annealing. Materials.

[51] Han M.K., Jin Y., Lee D.H., Kim S.J. (2017). Thermoelectric Properties of Bi₂Te₃: CuI and the Effect of Its Doping with Pb Atoms. Materials.

[52] Burkov A.T., Heinrich A., Konstantinov P.P., Nakama T., Yagasaki K. (2001). Experimental set-up for thermopower and resistivity measurements at 100–1300 K. Meas. Sci. Technol..

[53] Dörling B., Zapata-Arteaga O., Campoy-Quiles M. (2020). A setup to measure the Seebeck coefficient and electrical conductivity of anisotropic thin-films on a single sampl. Rev. Sci. Instrum..

[54] Asheghi M., Kurabayashi K., Kasnavi R., Goodson K.E. (2002). Thermal conduction in doped single-crystal silicon films. J. Appl. Phys..

[55] Périchon S., Lysenko V., Roussel P., Remaki B., Champagnon B., Barbier D., Pinard P. (2000). Technology and micro-Raman characterization of thick meso-porous silicon layers for thermal effect microsystems. Sens. Actuators A Phys..

[56] Sidhu L.S., Kosteski T., Zukotynski S. (1999). Infrared vibration spectra of hydrogenated, deuterated, and tritiated amorphous silicon. J. Appl. Phys..

[57] Lüpke G., Tolk N.H., Feldman L.C. (2003). Vibrational lifetimes of hydrogen in silicon. J. Appl. Phys..

[58] Singh V., Yu Y., Sun Q.C., Korgel B., Nagpal P. (2014). Pseudo-direct bandgap transitions in silicon nanocrystals: Effects on optoelectronics and thermoelectrics. Nanoscale.

[59] Boukai A.I., Bunimovich Y., Tahir-Khali J., Goddard W.A., Heath J.R. (2008). Silicon nanowires as efficient thermoelectric mate. Nature.

