# Supplementary material for: Effect of Nanographene Coating on the Seebeck Coefficient of Mesoporous Silicon
Source: Nanomaterials (Basel). 2023 Apr 1;13(7):1254. doi: 10.3390/nano13071254 (PMC10097016; doi:10.3390/nano13071254)
Supplement: Supplementary file 1 [file nanomaterials-13-01254-s001.zip › nanomaterials-2325083-supplementary.pdf]

Supplementary

Effect of Nanographene Coating on the Seebeck Coefficient of Mesoporous Silicon

Sibel Nar [1,2,3], Arnaud Stolz [1], Denis Machon [2,3,4], Eric Bourhis [5], Pascal Andreazza [5], Abderraouf Boucherif [2,3,*] and Nadjib Semmar [1,*]

1 Groupe de Recherches sur l'Énergétique des Milieux Ionisés, GREMI, Université d'Orléans, CNRS, 14 Rue d'Issoudun, 45067 Orleans Cedex 02
2 Laboratoire Nanotechnologies et Nanosystèmes (LN2) – CNRS IRL-3463, Université de Sherbrooke, 3000 Boulevard Université, Sherbrooke, Québec, J1K OA5, Canada
3 Institut Interdisciplinaire d'Innovation Technologique (3IT), Université de Sherbrooke, 3000 Boulevard de l'Université, Sherbrooke, Québec, J1K OA5, Canada
4 Université de Lyon, INSA Lyon, CNRS, École Centrale de Lyon, Université Claude Bernard Lyon 1, CPE Lyon, INL, UMR5270, 69621 Villeurbanne, France
5 Interfaces, Confinement, Matériaux et Nanostructures, ICMN, Université d'Orléans, CNRS, 1B, Rue de la Férollerie, 45071 Orleans Cedex 02
* Correspondence: abderraouf.boucherif@usherbrooke.ca (A.B.); nadjib.semmar@univ-orleans.fr (N.S.)

S1. Steps of CVD

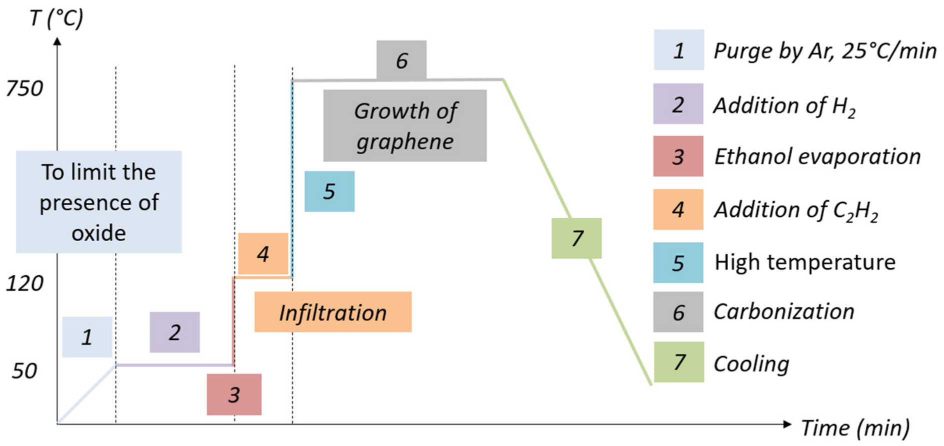

Figure S1. Steps of CVD for graphene coating of PSi, inspired by [23–24]

Step 1 is a purge of about ten minutes of argon (Ar) to limit the presence of oxide. Next, dihydrogen is introduced. The flow of hydrogen (H_2) is constant and is added to that of argon. The temperature is increased in step 3) to evaporate the ethanol up to 120 °C (boiling temperature of ethanol (~80 °C). The porous structure is filled with ethanol to preserve the sample and avoid its oxidation before deposition, hence the need for the evaporation step. Precursor insertion begins in step 4. Acetylene is added at a constant rate. The temperature is then increased to achieve growth. For mesoporous silicon, growth takes approximately 40 minutes. In the case of mesoporous germanium, growth takes ten minutes.

S2. Refractive index determination

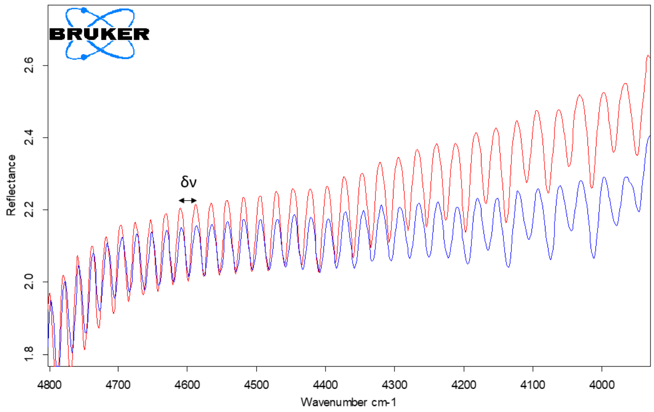

Figure S2. Refractive index determination of PSi by FTIR (red line for PSi-2 and blue line for PSi-3)

Table S1. Calculation of deviation and errors

Sample	Pores number (P_n) (1)	Pores number (P_n) (2)	Standard deviation σ_{Pn}
PSi-2	3621	3596	17.7
PSi-3	2932	2909	16.3
PSi-5	2557	2535	15.6
PSi-7	2197	2180	12.0
PSi-8	1412	1398	9.9

The analyzes were carried out twice in order to have an estimation of the error due to the image processing step.

S3. UPS spectra polynomial fit for Si

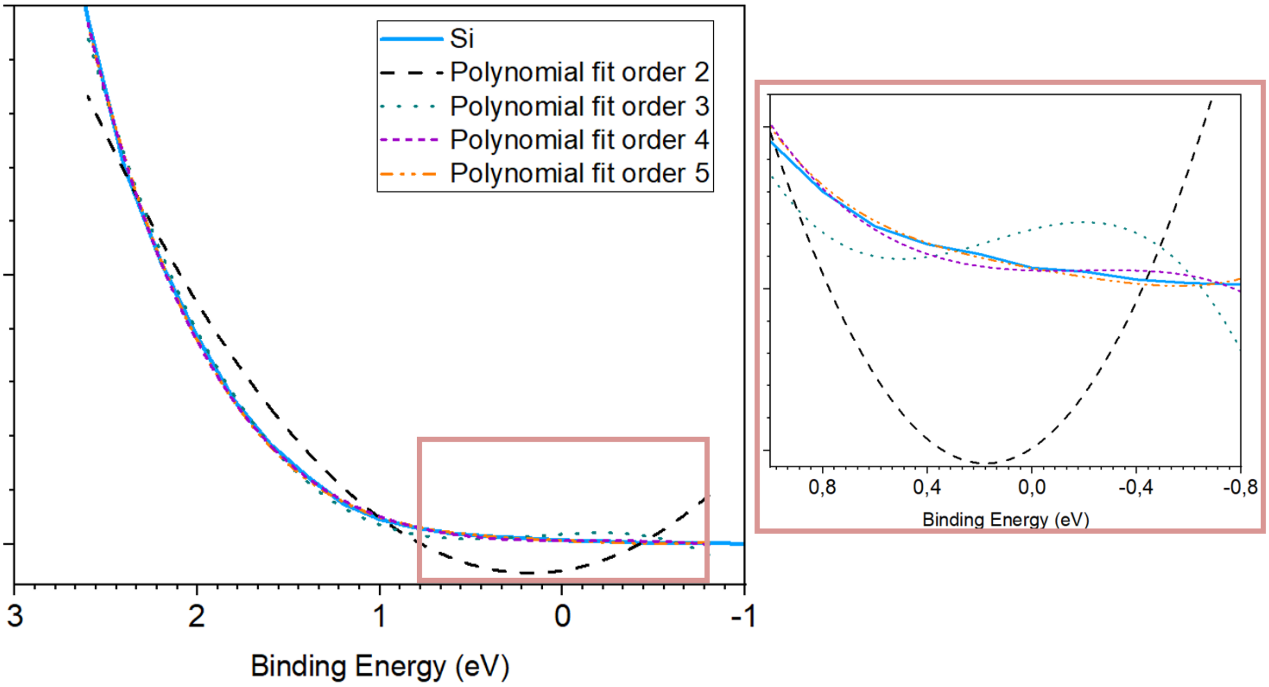

Figure S3. Methodology for fit and S value determination of Si by UPS

Table S2. Summary of polynomial fit characteristics.

Polynomial fit order	R^2	B_1
2	0.95301	535.8 ± 325.3
3	0.99722	199.2 ± 85.0
4	0.99951	149.2 ± 58.1
**5**	**0.99968**	**23.1 ± 13.7**

S4. Seebeck coefficient values

The analysis starts with the characterization of the thermoelectric properties of bismuth tellurium, a solid material with a thickness of 3 mm. Bi_2Te_3 is chosen for its thickness and known thermoelectric impact. A temperature gradient is imposed to evaluate the potential difference created in the presence of this material. Once these results have been obtained at different imposed temperatures, the potential variation is plotted as a function of the temperature variation. The small temperature variations allow the thermal stability to be noted and the actual Seebeck coefficients of the material to be derived. S values are shown below.

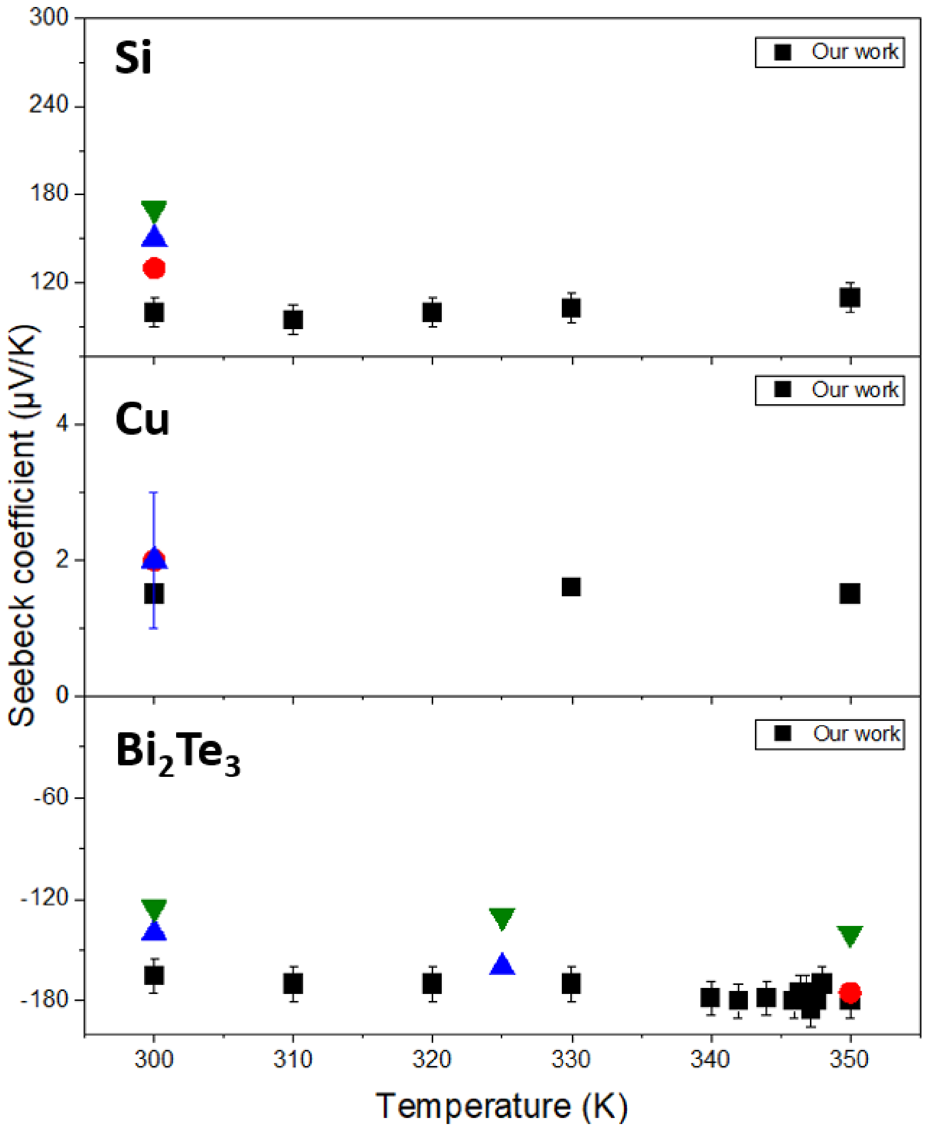

Figure S4. S values for Si [16–18], Bi_2Te_3 [49–51] and Cu [52,53]

This experiment is repeated for several temperatures to ensure that for small temperature variations the Seebeck values are similar and therefore repeatable. This will allow further thermoelectric measurements to be made on Cu, a thinner material, and on Si. Our thermoelectric device can therefore be used to measure the Seebeck coefficient of thin materials (of the order of a hundred microns) and low Seebeck coefficients, particularly in the case of Cu.

S5. COMSOL Multiphysics ® simulation

Since, we are in conduction, in a steady state, the essential parameter to take into account is the thermal conductivity (k) of the Si and PSi. For p-doped Si, k= 130 W/m.K has been estimated according to [54]. As for the k of PSi which is a good thermal insulator, it is considered equal to 1 W/m.K [31], [55]. The figure below illustrates a diagram of the considered system (Figure S4-a) as well as the simulation carried out (Figure S4-b). We observe the establishment of the thermal gradient between the two copper blocks in the PSi and Si. We impose a ΔT= 10K in order to visualize the temperature (T) distribution in both. According to the simulation, T decreasing is linked to PSi and the difference of T in the Si part is very small, which shows that the contribution of the S of Si does not disturb the measurement of S of PSi.

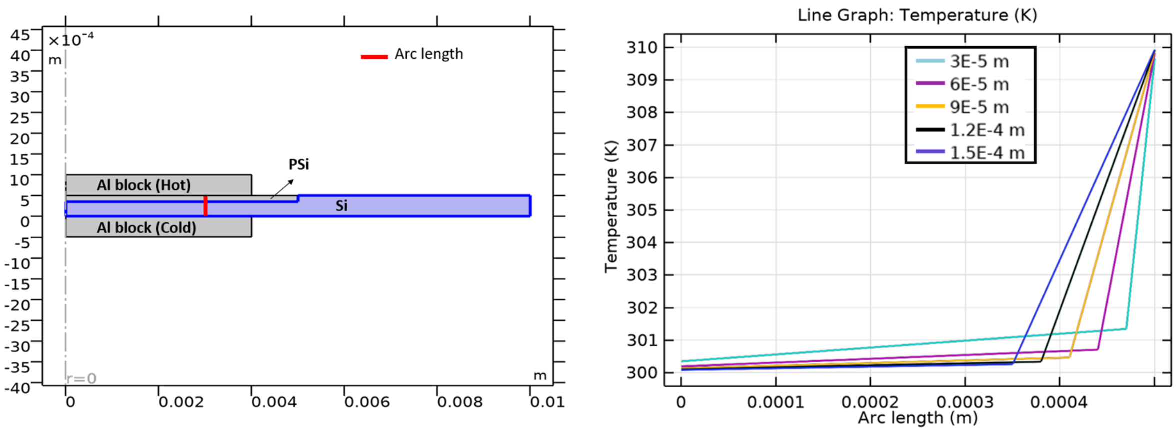

(**a**) (**b**)

Figure S5. (a) System studied under Comsol Multiphysics ® and (b) Establishment of the thermal gradient in the PSi and Si. The insert PSi thicknesses were selected as an example for the simulation study.
